# Astragaloside IV Improves the Barrier Damage in Diabetic Glomerular Endothelial Cells Stimulated by High Glucose and High Insulin

**DOI:** 10.1155/2022/7647380

**Published:** 2022-03-17

**Authors:** Tingting Zhao, Jiaye Tian, Tiancheng Xu, Xi Zhang, Qian Xiang, Jianfeng Xiong, Dingkun Gui, Youhua Xu

**Affiliations:** ^1^Faculty of Chinese Medicine, State Key Laboratory of Quality Research in Chinese Medicines, Macau University of Science and Technology, Avenida Wai Long, Taipa, Macao SAR, China; ^2^School of Chinese Medicines, Guangzhou University of Chinese Medicine, Guangzhou, China; ^3^Department of Nephrology, Shanghai Jiao Tong University Affiliated Sixth People's Hospital, Shanghai, China; ^4^Macau University of Science and Technology Zhuhai MUST Science and Technology Research Institute, Hengqin, Zhuhai, China; ^5^Department of Endocrinology, Zhuhai Hospital of Integrated Traditional Chinese and Western Medicine, Zhuhai, China

## Abstract

**Objective:**

To investigate the protective effect and mechanism of astragaloside IV (AS-IV) on damage in human glomerular endothelial cells (GEnCs) stimulated by high glucose and high insulin.

**Methods:**

The transwell method was used to detect the integrity of the cell barrier after AS-IV intervention in a high glucose and high insulin environment for 24 h; immunofluorescence and Western blot methods were used to detect the tight junction protein ZO-1 and claudin-5 expression; intracellular and extracellular 1*β* (IL-1*β*) and tumor necrosis factor *α* (TNF*α*) were determined by ELISA; expression and activation of AKT, p-AKT, GSK3*α*/*β*, and p-GSK3*α*/*β* were evaluated by Western blot.

**Results:**

The results showed that AS-IV had a significant protective effect on the cell barrier of GEnCs. High glucose or insulin inhibited cell viability in a concentration-dependent manner. High glucose or insulin significantly inhibited glucose uptake and promoted release of reactive oxygen species in GEnCs. Administration with AS-IV dramatically preserved viability of the cells; moreover, the expression of intracellular tight junction proteins was upregulated, inflammatory cytokines including IL-1*β* and TNF*α* were decreased, and the AKT-GSK3 pathway participated in modulation of AS-IV in GEnCs cells.

**Conclusion:**

We found in the present study that AS-IV can preserve filtration barrier integrity in glomerular endothelial cells under diabetic settings, its effects on increasing the cell energy metabolism and cell viability, inhibiting inflammation and oxidative stress damage, and enhancing tight junction between cells play a role in it; and the intracellular signaling pathway AKT-GSK modulated the above function. Our present finding supplied a new understanding towards development of DN and provided an alternative method on ameliorating DN.

## 1. Introduction

Diabetes mellitus (DM) is a common metabolic disease, and hyperglycemia is a clinical feature. Hyperglycemia is caused by defective insulin secretion or impaired biological effects or both [1]. Chronic hyperglycemia can lead to chronic impairment of various tissues, especially microvascular tissues. It is reported that even if blood pressure and glucose control are met, 30%–40% of diabetic patients will eventually develop into diabetic nephrology (DN), which is the leading cause of end stage renal disease (ESRD) [2]. More importantly, with the disease progression, renal structure and dysfunction are difficult to reverse [3]. Currently, the lack of early and effective interventions has become the bottleneck problem in treatment of DN [4].

Proteinuria is the main clinical feature of DN and an independent risk factor for the progression of DN. It is mainly attributed to the impairment of the glomerular filtration barrier. Glomerular endothelial cells (GEnCs) are an important part of the glomerular filtration barrier and play a pivotal role in regulating the glomerular filtration. GEnCs dysfunction occurs in the early stages of DN and is characterized by impaired endothelial glycocalyx, inflammatory phenotype, mitochondrial damage, and oxidative stress [5, 6]. Recent advances suggest that GEnC is an ideal target on delaying development of DN [5].

Astragaloside IV (AS-IV) is one of the effective active ingredients of *Astragalus*. It was reported that AS-IV has broad effects against disease, such as antioxidation [7,8], anti-inflammation [9,10], and preserving heart function [11]. A most recent report demonstrated that AS-IV can reduce the kidney damage in DN and membranous nephropathy [12]. However, it is unclear whether AS-IV may directly prevent and ameliorate T2D-induced DN and how AS-IV may regulate this process. To this end, in vitro experiments were carried out in the current study, and the underlying mechanism was studied. Our present study and finding may provide new insights on the role of AS-IV in the intervention of DN.

## 2. Materials and Methods

### 2.1. Materials

Human glomerular endothelial cells (GEnCs) were purchased from Fenghui Biological. Astragaloside IV was supplied by China Bailingwei Technology Co., Ltd. (CAS#83207-58-3). Metformin was supplied by Matemi (USA). Glucose, insulin, and 70 kDa dextran were derived from Sigma (USA). Detection kits for superoxidase dismutase (SOD), malondialdehyde(MDA), intracellular and extracellular 1*β* (IL-1*β*), and tumor necrosis factor *α* (TNF*α*) were purchased from Nanjing Jiancheng (Nanjing, China). Transwell was derived from Corning (USA). ECM medium was provided by ScienCell (USA). Primary antibodies for protein kinase B (AKT), phosphoprotein kinase B (p-AKT), glycogen synthase kinase 3 *α*/*β* (GSK3*α*/*β*), phosphoglycogen synthase kinase 3 *α*/*β* (p-GSK3*α*/*β*), claudin-5, and zonula occluden-1 (ZO-1) were from Santa Cruz Biotechnology (USA). The other reagents were from commercial sources.

### 2.2. Cell Culture

GEnCs were cultured in a cell culture medium containing 5.6 mmol/L glucose, 5% fetal bovine serum, 1% penicillin-streptomycin, and 1% endothelial cell growth factor at 37°C and 5% CO_2_. Cell culture medium was changed daily, and the cells were passaged every 2 days.

### 2.3. MTT Assay for Cell Viability

GEnCs cells at the logarithmic phase are placed into the 96-well. After drug incubation as indicated, MTT (5 mg/mL) solution was added into the culture system, and the plate was further incubated at 37°C for 4 h. Finally, the culture medium was replaced by DMSO, and cell viability was assayed on a microplate reader under the wavelength of 490 nm.

### 2.4. Reactive Oxygen Species Detection

Cells at the logarithmic phase are placed into a six-well cell culture plate at the density of 3 × 10^5^ cells/mL. After being administrated with drugs, the culture medium was replaced by 2-NBDG dye (30 *μ*M) or DCFH dye solution (10 *μ*M). Twenty minutes later, the fluorescent dye solution was discarded, and level of reactive oxygen species was observed under a fluorescence microscope (OLYMPUS, Japan).

### 2.5. ELISA Kit Detection

The levels of MDA (A003-1, Nanjing Jiancheng), SOD (A001-3, Nanjing Jiancheng), IL-1*β* (H002, Nanjing Jiancheng), and TNF*α* (H052-1, Nanjing Jiancheng) in GEnCs cells were detected according to the protocols supplied from the manufacturer.

### 2.6. Establishment of GEnCs Monocellular Barrier

Cells at the logarithmic phase are placed into the upper chamber of the transwell at a concentration of 3 × 10^5^/ml. The resistance value of each well was measured and recorded every other day until the 21^st^ day when the cells reach their tightest connection state.

Once the monocellular barrier model was successfully constructed, cells were treated with vehicle, H-Glu (30 mM) + Ins (1 *μ*M), H-Glu + Ins + AS-IV (100 *μ*g/mL), or H-Glu + Ins + Met (0.5 mM). Twenty-four hours later, the resistance value of each group was recorded. Thereafter, FITC-dextran (70 kDa) at a final concentration of 2 mg/ml was added into the upper chamber of each well at dark atmosphere, content of dextran in the lower chamber was detected under the wavelengths of excitation light and absorption light at 492 nm and 518 nm by a microplate reader at 0 h, 1 h, 2 h, and 3 h, and the dextran leakage coefficient at different time points was finally obtained.

### 2.7. Immunofluorescence Assay

Cells were treated according to the method described in “2.4”. Twenty-four hours after drug intervention, cells were fixed with 4% paraformaldehyde for 15 minutes. The cells were then blocked with 5% BSA and incubated with primary antibodies including ZO-1 (1 : 200) and claudin-5 (1 : 200) at 4°C overnight. After being gently washed with PBS, the cells were incubated with FITC-conjugated secondary antibodies. The nucleus was stained with DAPI. Finally, relative expression of proteins was observed under a confocal laser scanning microscope.

### 2.8. Western Blot Analysis (WB)

Cells were treated according to the method described in “2.4”. Twenty-four hours after drug intervention, proteins were extracted with RIPA, and protein concentration was determined with the BCA protein quantification kit. Thereafter, proteins were separated by a 10% SDS-PAGE gel for gel electrophoresis; then, the sample was transferred to PVDF membrane and blocked with BSA solution. The membrane was incubated with primary antibodies including AKT (1 : 1000), GSK3 *α*/*β* (1 : 1000), P-GSK3 *α*/*β* (1 : 1000), GAPDH (1 : 1000), ZO-1 (1 : 1000), or claudin-5 (1 : 1000) overnight at 4°C and then was further incubated with secondary antibody (1 : 5000) for 1 h at room temperature. The infrared fluorescence scanning imaging system was used for protein expression measurement and analysis.

### 2.9. Statistical Analysis

The data were statistically analyzed with GraphPad Prism 7.00. The data are expressed by mean ± standard deviation, and the comparison of the means among groups is performed by a one-way analysis of variance. *P* < 0.05 or less is regarded to be statistically significant.

## 3. Results

### 3.1. Construction of the Diabetic Model in Glomerular Endothelial Cells (GEnCs)

**Figure 1:**
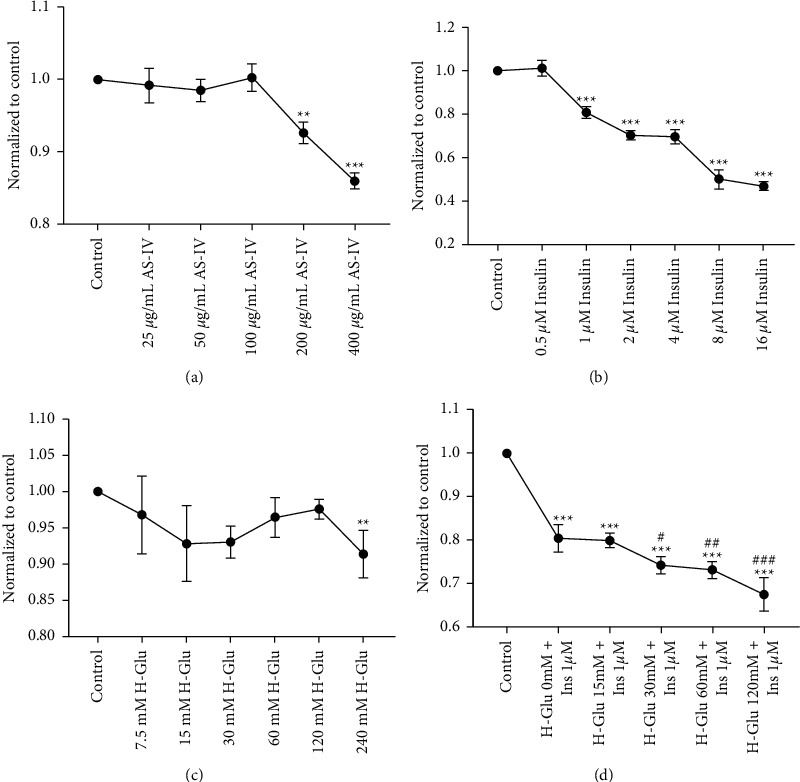
GEnCs cell viability assay by MTT. (a) Astragaloside IV (AS-IV), (b) insulin (Ins), (c) high glucose (H-Glu), and (d) high glucose + high insulin (H-Glu + Ins) concentration-dependently inhibited GEnCs cell viability;  ^*∗*^*p* < 0.05,  ^*∗∗*^*p* < 0.01,  ^*∗∗∗*^*p* < 0.001 vs. control; ^#^*p* < 0.05, ^##^*p* < 0.01, ^###^*p* < 0.001 vs. H-Glu 0 mM + Ins 1 *μ*M.

To elucidate the effects and mechanism of AS-IV on the glomerular filtration barrier, we first constructed a diabetic model in GEnCs and evaluated influence of AS-IV on viability of the cells. As shown in Figures 1(a)-1(c), cell viability was inhibited by AS-IV, glucose, or insulin in a concentration-dependent manner after 24 h incubation. Insulin resistance and hyperglycemia are two characteristics of diabetes. To this end, cells were further incubated with insulin + high-glucose, and we found cell viability was further decreased (Figure 1(d)).

**Figure 2:**
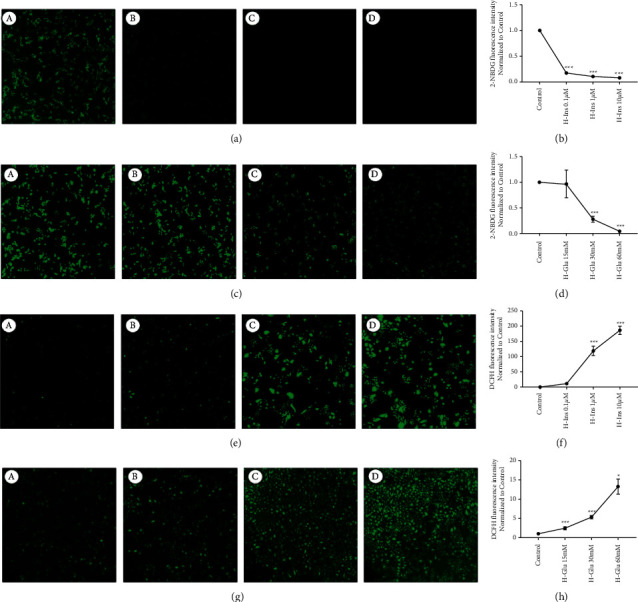
Cell glucose uptake ability and ROS content assay under high insulin + glucose condition. (A) High insulin concentration-dependently inhibited cell glucose uptake. (a)–(d) The concentration of insulin at 0, 0.1, 1, and 10 *μ*M. (B) 2-NBDG fluorescence analyzed for data (A). (C) High glucose concentration-dependently inhibited cell glucose uptake. (a)–(d) The concentration of insulin at 0, 15, 30, and 60 mM. (D) 2-NBDG fluorescence analyzed for data (C). (e) High insulin concentration-dependently promoted cell oxidative stress. (a)–(d) The concentration of insulin at 0, 0.1, 1, and 10 *μ*M. (f) 2-NBDG fluorescence analyzed for data (e). (g) High glucose concentration-dependently promoted cell oxidative stress. (a)–(d) The concentration of insulin at 0, 15, 30, 60 mM. (H) 2-NBDG fluorescence was analyzed for data (g); *∗p* < 0.05, *∗∗p* < 0.01,  ^*∗∗∗*^*p* < 0.01 vs. control.

To further validate functional changes of insulin and high glucose, glucose uptake as well as oxidative stress were determined. We found glucose uptake (Figures 2(a)–2(d)) was significantly reduced with concentration increase of glucose or insulin, accompanied with level of reactive oxygen species (ROS) increment (Figures 2(e)–2(h)).

Converging with Figures 1 and 2, we applied 30 mM high glucose and 1 *μ*M high insulin to construct the diabetic model in GEnCs, and concentration of 100 *μ*g/mL for AS-IV was used to evaluate its effects on diabetic-GEnCs.

### 3.2. Astragaloside IV Inhibited the Expression of Inflammatory Cytokines and Ameliorated Oxidative Stress Damage

Inflammation and oxidative stress are two typical phenomena in which they will directly inhibit viability of GEnCs. In the present study (Table 1), we found both inside and outside of inflammatory cytokines including TNF*α* and IL-1*β* were significantly elevated by high glucose and high insulin incubation, while AS-IV administration inhibited their secretion to the level that is comparable to normal. Similar results were observed in SOD and MDA expressions that AS-IV increased antioxidant SOD while decreased prooxidant MDA levels.

### 3.3. Astragaloside IV (AS-IV) Protected Monocellular Barrier of GEnCs Cells

**Figure 3:**
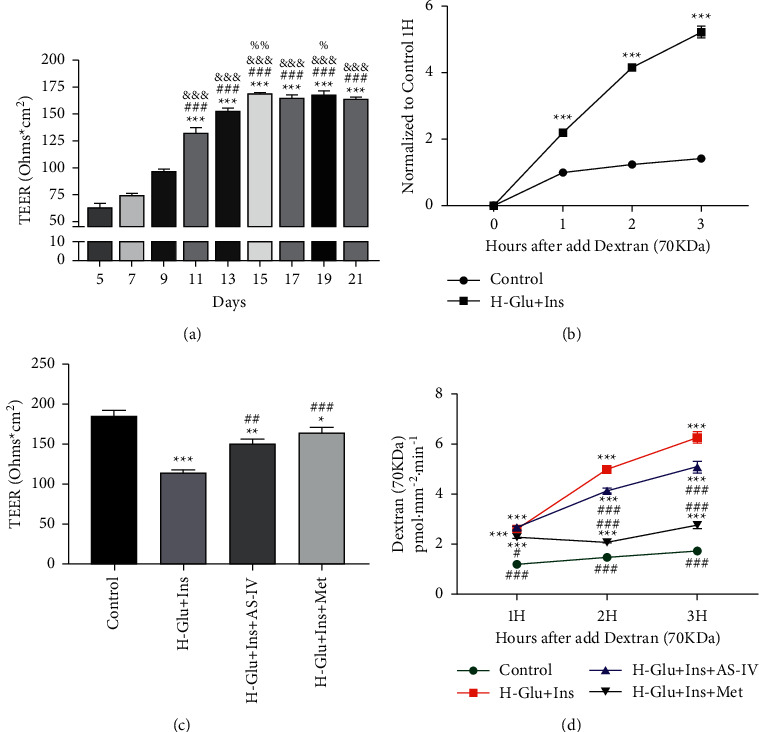
Effects of astragaloside IV(AS-IV) on barrier integrity of GEnCs cells stimulated by high insulin and high glucose. (a) The assay of transendothelial electrical resistance (TEER). In a normal environment, the maximum TEER value in GEnCs was within days 15–21;  ^*∗∗∗*^*p* < 0.001 vs.7 d; ^###^*p* < 0.001 vs.9 d; ^&&&^*p* < 0.001 vs.11 d; ^%^*p* < 0.05, ^%%^*p* < 0.01 vs. 13 d. (b) After high glucose and high insulin stimulation, dextran in the lower was significantly increased,  ^*∗∗∗*^*p* < 0.001 vs. control. (c) After drug stimulation, the TEER value was significantly reduced;  ^*∗*^*p* < 0.05,  ^*∗∗*^*p* < 0.01,  ^*∗∗∗*^*p* < 0.001 vs. control; ^#^*p* < 0.05, ^##^*p* < 0.01, ^###^*p* < 0.001 vs. H-Glu + Ins. (d) AS-IV treatment reduced leakage of dextran (70 kD) from the monocellular barrier,  ^*∗∗∗*^*p* < 0.001 vs. control; ^###^*p* < 0.001 vs. H-Glu + Ins.

To analyze influence of AS-IV on the endothelial barrier, we first constructed a monocellular barrier on upper chamber of transwell according to the method described previously [13]. We found the TEER reached a maximum value on day 21 (Figure 3(a)). After successful construction of the monocellular barrier, the cells were further induced by high glucose and high insulin; we found a significant barrier leakage in which dextran (70 kDa) in the lower chamber was strikingly increased by as much as 5 times compared with that of control (*p* < 0.001) (Figure 3(b)). In line with our expectation, AS-IV administration significantly enhanced the barrier integrity (Figure 3(c)) and decreased the barrier leakage of dextran (Figure 3(d)).

### 3.4. Astragaloside IV Upregulated Expression of Tight Junction Protein in GEnCs

**Figure 4:**
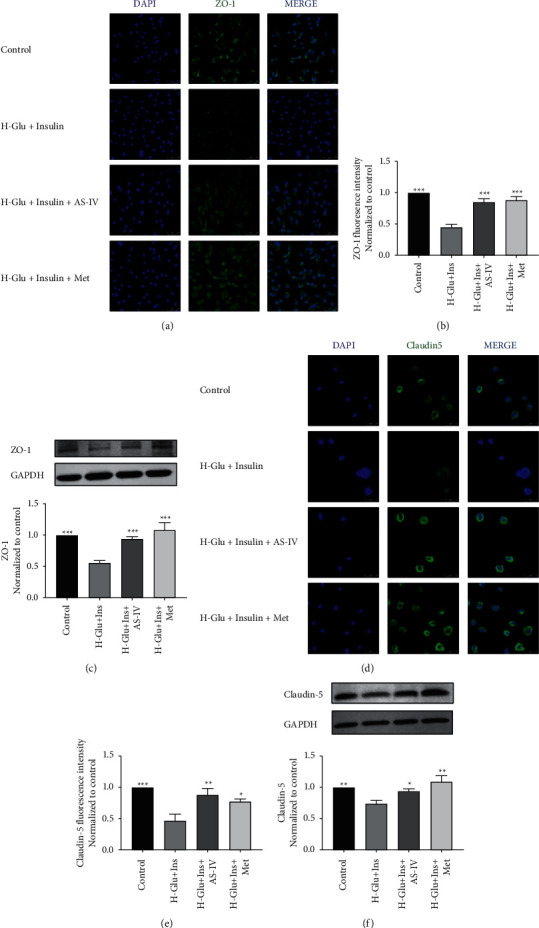
Effects of astragaloside IV on tight junction proteins in GEnCs cells. Expressions of (a) ZO-1 and (d) claudin-5 were determined by immunofluorescence assay under the laser scanning confocal microscope. Relative fluorescence intensities of (b) ZO-1 and (e) claudin-5 were determined by Image-J software. WB detection for proteins expression and quantitative analysis of ZO-1 (c) and claudin-5 (f);  ^*∗*^*p* < 0.05,  ^*∗∗*^*p* < 0.01,  ^*∗∗∗*^*p* < 0.001 vs. H-Glu + Ins.

Normal expression of tight junction proteins plays a central role in maintaining function of biological barriers. By immunofluorescence and WB, we found expressions of ZO-1 (Figures 4(a)–4(c)) and claudin-5 (Figure 4(d)–4(f)) were significantly inhibited in the diabetic model, while both AS-IV and metformin treatment upregulated their expression.

### 3.5. AKT-GSK3 Pathway Is Involved in AS-IV-Mediated Protection of GEnCs Filtration Barrier

**Figure 5:**
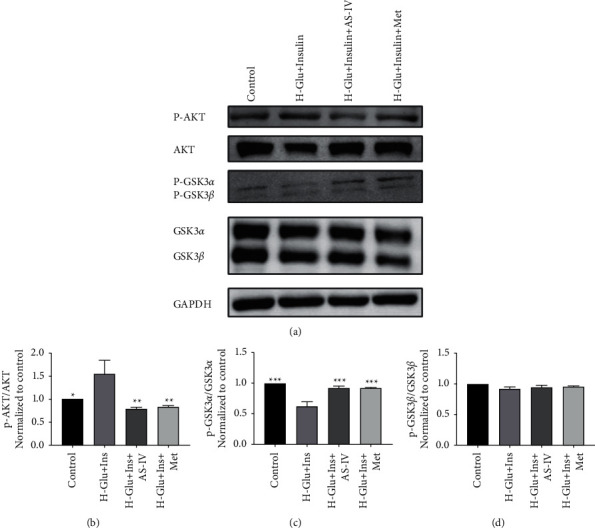
Akt/GSK3*α*/*β* signaling pathway evaluation. (a) Expression of p-Akt, Akt, p-GSK3*α*/*β*, GSK3*α*/*β*, and GAPDH determined by Western blot. Relative expression of the proteins was assayed by Image-J software, and ratio of (b) p-Akt/Akt, (c) p-GSK3*α*/GSK3*α*, and (d) p-GSK3*β*/GSK3*β* was obtained;  ^*∗*^*p* < 0.05,  ^*∗∗*^*p* < 0.01,  ^*∗∗∗*^*p* < 0.001 vs. H-Glu + Ins.

To further explore the mechanism of AS-IV on protecting GEnCs, expression and activation of intracellular signaling proteins were detected by WB. We found that diabetes significantly increased ratio of p-AKT/AKT while decreased p-GSK3*α*/GSK3*α*; and AS-IV administration strikingly reversed effects of high glucose + high insulin on AKT and GSK3 (Figure 5).

## 4. Discussion

Glomerular filtration barrier dysfunction is always a central event that contributes to proteinuria. In the present study, we demonstrated that astragaloside IV (AS-IV) possessed a significant effect on preserving viability as well as filtration barrier integrity in glomerular endothelial cells.

Currently, a typical strategy in clinics to treat DN is application of renin-angiotensin-aldosterone (RAS) drugs [14]. However, the long-term effect is limited, and there is a high demand for effective interventions in the early and mid-term of DN. *Astragalus* is the root of *Astragalus mongolicus* and *Astragalus membranaceus*. It has been used as medicine for more than 2,000 years. Astragaloside IV (AS-IV) is the main active ingredient extracted from medicinal *Astragalus*. Studies have found that AS-IV can enhance the body's immune function, antioxidant damage, anti-inflammation, regulate energy metabolism, protect nerve functions, and improve endothelial cells viability; an in vivo study found that AS-IV plays an important role in the function of neovascularization and diuresis [15–17]. Wen and colleagues demonstrated that AS-IV could protect renal tubular damage in DN both in vivo and in vitro [18]. More importantly, studies indicated that application of *Astragalus* can significantly delay the progression of DN [19, 20]. However, the direct effect and mechanism of AS-IV on glomerular endothelial cells are still largely unknown.

The normal performance of the barrier function mainly depends on the integrity of tight junction proteins [21]. ZO-1 and claudin-5 are important components of the tight junctions between endothelial cells. In this study, we applied high glucose accompanied with high insulin and successfully constructed a filtration barrier dysfunction model. We found both the viability and tight junction proteins are inhibited under diabetes settings, and AS-IV administration significantly increased viability of the cells and reduced barrier leakage.

Recently, studies [5] have shown that GEnCs dysfunction occurs in the early stages of focal segmental glomerulosclerosis and DN and is characterized by damage to the endothelial glycocalyx, inflammatory phenotype, mitochondrial damage, oxidation stress, and abnormal cell signal transduction. In our previous in vivo and in vitro studies [22], we also demonstrated that inhibition of inflammation and oxidative stress is helpful to preserve cell viability and against diabetic damage. In consistent with previous reports, we found in the present study that AS-IV significantly reduced secretion of inflammatory cytokines and ameliorated oxidative stress damage induced by high glucose and high insulin.

AKT and GSK3 play an important role in modulating cell genesis as well as the energy metabolism [23]. It was found that insulin resistance will induce activation of AKT which will further inhibit GSK3 [24, 25]. Zhou and colleagues [26] found that *Astragalus* can inhibit the proliferation of breast cancer cells, and its mechanism was related to the downregulation of AKT phosphorylation. In the present study, AS-IV was shown with significant effects on inhibiting activation of AKT, and increasing activation of GSK in that activation form of GSK3*α*, but not p-GSK3*α*, was increased; therefore, AS-IV may function via increasing glucose uptake of GEnCs and enhancing the energy metabolism via the GSK3*α* pathway.

**Figure 6:**
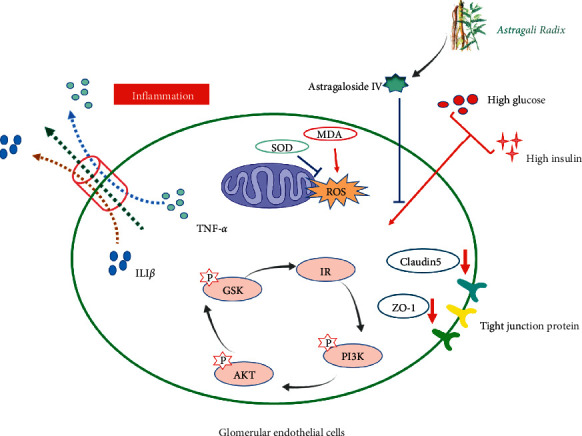
The mechanism of astragaloside IV on the barrier integrity of diabetic glomerular endothelial cells.

In conclusion, we found in the present study that AS-IV can preserve filtration barrier integrity in glomerular endothelial cells under diabetic settings, its effects on increasing the cell energy metabolism and cell viability, inhibiting inflammation and oxidative stress damage, and enhancing tight junction between cells play a role in it; and intracellular signaling pathway AKT-GSK modulated the above function (Figure 6). Our present finding supplied a new understanding towards development of DN, and provided an alternative method on ameliorating DN.

## Figures and Tables

**Table 1:** Effects of astragaloside IV on inflammation and oxidative stress factors.

Group	TNF*α* (intracellular), ng/L	TNF*α* (extracellular), ng/L	IL-1*β* (intracellular), ng/L	IL-1*β* (extracellular), ng/L	SOD, U/mg prot	MDA, nmol/mg prot
Control	398.61 ± 3.95	352.24 ± 1.91	659.89 ± 2.79	644.23 ± 7.11	271.55 ± 4.66	3.93 ± 0.21
H-Glu + Ins	461.39 ± 9.1 ^*∗∗∗*^	505.92 ± 9.53 ^*∗∗∗*^	814.75 ± 8.54 ^*∗∗∗*^	754.77 ± 3.48 ^*∗∗∗*^	143.53 ± 26.16 ^*∗∗∗*^	8.48 ± 0.25 ^*∗∗∗*^
H-Glu + Ins + AS-IV	412.8 ± 5.38^###^	376.09 ± 3.31^###^	735.3 ± 2.84^###^	687.3 ± 6.84^###^	254.66 ± 5.31^###^	5.41 ± 0.18^###^

^*∗∗∗*^*p* < 0.01 vs. control; ^###^*p* < 0.01 vs. H-Glu + Ins.

## Data Availability

The data used to support the findings of this study are available from the corresponding author upon request.
